# Pemafibrate Prevents Retinal Dysfunction in a Mouse Model of Unilateral Common Carotid Artery Occlusion

**DOI:** 10.3390/ijms22179408

**Published:** 2021-08-30

**Authors:** Deokho Lee, Yohei Tomita, Heonuk Jeong, Yukihiro Miwa, Kazuo Tsubota, Kazuno Negishi, Toshihide Kurihara

**Affiliations:** 1 Laboratory of Photobiology, Keio University School of Medicine, Tokyo 160-8582, Japan; deokholee@keio.jp (D.L.); yohei.tomita@childrens.harvard.edu (Y.T.); jeong.h@keio.jp (H.J.); yukihiro226@gmail.com (Y.M.); 2 Department of Ophthalmology, Keio University School of Medicine, Tokyo 160-8582, Japan; kazunonegishi@keio.jp; 3 Department of Ophthalmology, Boston Children’s Hospital, Harvard Medical School, Boston, MA 02115, USA; 4 Animal Eye Care, Tokyo Animal Eye Clinic, Tokyo 158-0093, Japan; 5 Tsubota Laboratory, Inc., Tokyo 160-0016, Japan; tsubota@tsubota-lab.com

**Keywords:** common carotid artery occlusion, electroretinography, fibroblast growth factor 21, pemafibrate, peroxisome proliferator-activated receptor alpha, retinal ischemia

## Abstract

Cardiovascular diseases lead to retinal ischemia, one of the leading causes of blindness. Retinal ischemia triggers pathological retinal glial responses and functional deficits. Therefore, maintaining retinal neuronal activities and modulating pathological gliosis may prevent loss of vision. Previously, pemafibrate, a selective peroxisome proliferator-activated receptor alpha modulator, was nominated as a promising drug in retinal ischemia. However, a protective role of pemafibrate remains untouched in cardiovascular diseases-mediated retinal ischemia. Therefore, we aimed to unravel systemic and retinal alterations by treating pemafibrate in a new murine model of retinal ischemia caused by cardiovascular diseases. Adult C57BL/6 mice were orally administered pemafibrate (0.5 mg/kg) for 4 days, followed by unilateral common carotid artery occlusion (UCCAO). After UCCAO, pemafibrate was continuously supplied to mice until the end of experiments. Retinal function (a-and b-waves and the oscillatory potentials) was measured using electroretinography on day 5 and 12 after UCCAO. Moreover, the retina, liver, and serum were subjected to qPCR, immunohistochemistry, or ELISA analysis. We found that pemafibrate enhanced liver function, elevated serum levels of fibroblast growth factor 21 (FGF21), one of the neuroprotective molecules in the eye, and protected against UCCAO-induced retinal dysfunction, observed with modulation of retinal gliosis and preservation of oscillatory potentials. Our current data suggest a promising pemafibrate therapy for the suppression of retinal dysfunction in cardiovascular diseases.

## 1. Introduction

Ocular ischemic syndrome (OIS) is a vision-threatening disease caused by carotid artery stenosis or occlusion [1]. The first case was reported in 1963 as a disease associated with internal carotid artery occlusion [2]. About 7.5 cases per million are annually diagnosed with OIS [3]. It is most common in old males, and patients with underlying diabetes, hypertension, and hyperlipidemia are more likely to have this disease. Atherosclerosis has also been known to be one of the most common causes for the development of OIS [4]. Besides, carotid artery dissection, giant cell arteritis, and trauma can have high chances to cause OIS [5,6,7,8,9,10]. Unfortunately, there is no current effective treatment in OIS. Moreover, precise mechanisms of OIS have not been fully unraveled yet.

Experimental murine models of carotid artery occlusion have been applied to study OIS [11,12,13,14,15,16,17,18]. From an anatomical point of view, the retina is supplied with oxygen/blood from the ophthalmic artery, one of the internal carotid artery’s branches of the common carotid artery. In this regard, occlusion of the carotid artery can cause retinal ischemia leading to vision loss [19,20]. There have been several ways of developing murine models of carotid artery occlusion depending on the species. Two common carotid arteries could be occluded to induce retinal ischemia in rats. As the circle of Willis in rats is well-structured, the rats which receive bilateral common carotid artery occlusion (BCCAO) could be developed as experimental models of retinal ischemia [11,12,13,15]. Two common carotid arteries could not be occluded in mice because of a high rate of mouse death (almost 100%) as they may have a lack of posterior communicating arteries in the circle of Willis [17,21,22]. Therefore, bilateral common carotid artery stenosis (BCCAS) has been alternately attempted to induce severe retinal ischemic injuries in mice [14]. However, the concern about a high rate of death during and after BCCAO or BCCAS in rats or mice has not been solved in that the experimental models still die easily. Hence, unilateral common carotid artery occlusion (UCCAO) has been tried and developed in mice for studying retinal ischemia more stably [17,18,23]. Even though several phenotypes for retinal ischemia have been described [17,18,23], a rescue for retinal ischemia has not been considerably studied in this model. In this regard, the development of a cure for retinal ischemia in this model could be intriguing.

Peroxisome proliferator-activated receptor alpha (PPARα) is a well-known drug against hyperlipidemia. This agent can potentially reduce triglyceride levels and increase high-density lipoprotein cholesterol (HDL-C) levels [24]. The Fenofibrate Intervention and Event Lowering in Diabetes (FIELD) and The Action to Control Cardiovascular Risk in Diabetes (ACCORD) eye studies showed that fenofibrate, a well-known PPARα agonist, reduced the need for laser therapy and progression of diabetic retinopathy [25,26]. Thus, this drug was recently approved for preventing diabetic retinopathy in Australia. Several studies have shown that fenofibrate has therapeutic effects on retinal diseases in animal models [27,28,29]. However, fenofibrate may potentially induce renal dysfunction, and it may cause rhabdomyolysis when administered with a statin. Thus, clinicians needed to take care of this part when they prescribed fenofibrate for diabetic patients with renal dysfunction.

Pemafibrate, a novel selective PPARα modulator (SPPARMα), has been developed as a therapeutic agent against hyperlipidemia to reduce this side effect. Our previous study showed that pemafibrate might prevent pathological neovascularization in a murine model of oxygen-induced ischemic retinopathy and preserve retinal function in a streptozotocin-induced diabetic mouse model [30,31]. Another group showed that pemafibrate might prevent retinal inflammation and vascular leakage in a rat’s diabetic model and prevent apoptosis in the ganglion cells damaged by N-methyl-D-aspartate (NMDA)-induced excitotoxicity [32,33]. Taken together, we assumed that pemafibrate could be used for neuroprotection against various retinal ischemic injuries.

In this study, we aimed to investigate the protective effects of pemafibrate in a murine model of retinal ischemia induced by UCCAO, which resembles OIS.

## 2. Results

### 2.1. Suppression of Retinal Dysfunction by Pemafibrate Administration in a Mouse Model of UCCAO-Induced Retinal Ischemia

**Figure A1:**
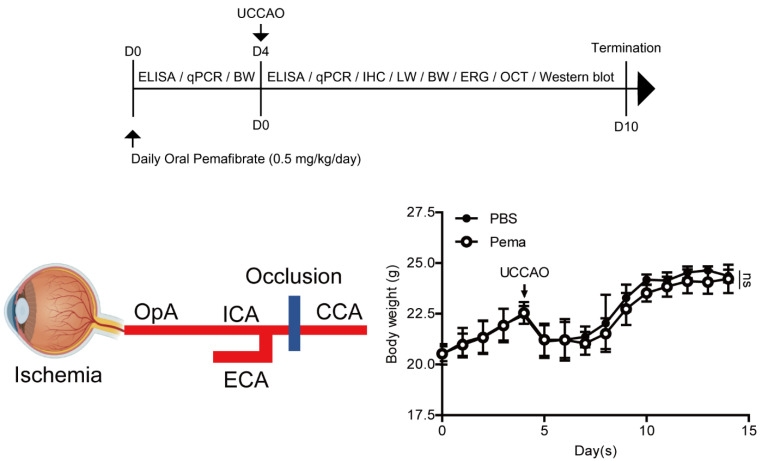
General monitoring for adult mice after consecutive oral administration of pemafibrate. A schematic illustration shows oral administration of pemafibrate (0.5 mg/kg/day) to mice and a time point of the UCCAO surgery and experiments followed. ELISA; enzyme-linked immunosorbent assay, qPCR; quantitative PCR, BW; body weight, IHC; immunohistochemistry, LW; liver weight, ERG; electroretinography, OCT; optical coherence tomography, UCCAO; unilateral common carotid artery occlusion. A schematic illustration of retinal ischemia induction by UCCAO. Retinal ischemia could be induced by occlusion (a blue bar) of the common carotid artery (CCA) as the ophthalmic artery (OpA) is originated from the internal carotid artery (ICA) of CCA. ECA; external carotid artery. Quantitative analyses (*n* = 5–10 per group) showed that the body weight of mice became lower after UCCAO. There was no dramatic difference in the body weight between pemafibrate-administered mice and PBS-administered mice. However, mice showed a slight decrease in the body weight after consecutive administration of pemafibrate without any statistical significance. *p* > 0.05. The data were analyzed using two-way ANOVA followed by a Bonferroni post hoc test. The data were presented as mean ± standard deviation. Pema; pemafibrate. ns; not significant.

According to our timeline of experiments, pemafibrate was orally administered to adult male mice (0.5 mg/kg/day) for 4 days before UCCAO (Figure A1). The administration of pemafibrate did not significantly change the body weight of adult male mice. After 4 days of oral administration of pemafibrate, retinal ischemia was induced by occlusion of the right common carotid artery which is connected to the internal carotid artery stretched toward the ophthalmic artery (Figure A1). We found that the body weight of adult male mice dramatically decreased 1 day after UCCAO (Figure A1). Pemafibrate (0.5 mg/kg/day) was consecutively supplied to UCCAO-operated mice, and we found that administration of pemafibrate did not dramatically change the body weight of UCCAO-operated mice. However, there was a slightly decreasing tendency in the body weight of UCCAO-operated mice after continuous oral administration of pemafibrate.

**Figure 1:**
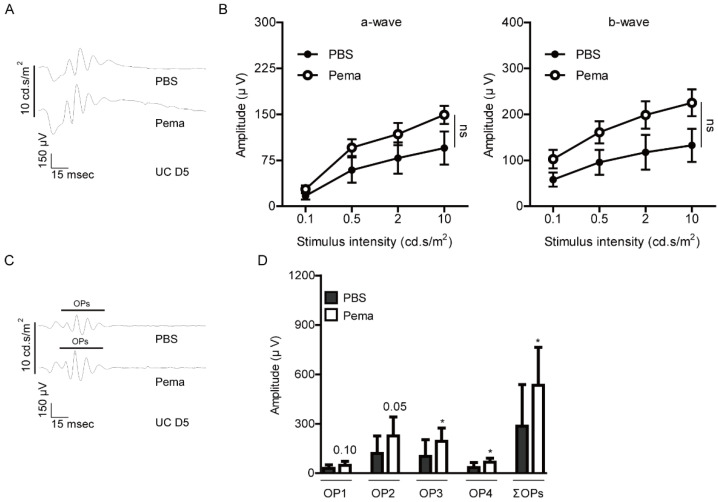
Protective effects of pemafibrate against retinal dysfunction on day 5 after UCCAO. (**A**,**B**) Representative waveforms (10 cd·s/m^2^) of a-and b-waves and quantitative analyses (*n* = 9–10 per group) showed that oral administration of pemafibrate had a slight suppressing tendency in a reduction in the amplitudes of a-wave and b-wave in the UCCAO-operated eye 5 days after UCCAO. The data were analyzed using two-way ANOVA followed by a Bonferroni post hoc test. The data were presented as mean ± standard error of the mean. (**C**,**D**) Representative waveforms (10 cd·s/m^2^) of oscillatory potentials (OPs) and quantitative analyses showed that pemafibrate significantly suppressed reduction in the amplitudes of OPs (OP1, OP2, OP3, and ΣOPs) in UCCAO-induced retinal ischemic mice (*n* = 9–10 per group). * *p* < 0.05. The data were analyzed using two-tailed Student’s *t*-test. The data were presented as mean ± standard deviation. Pema; pemafibrate. UC; unilateral common carotid artery occlusion. ns; not significant.

**Figure 2:**
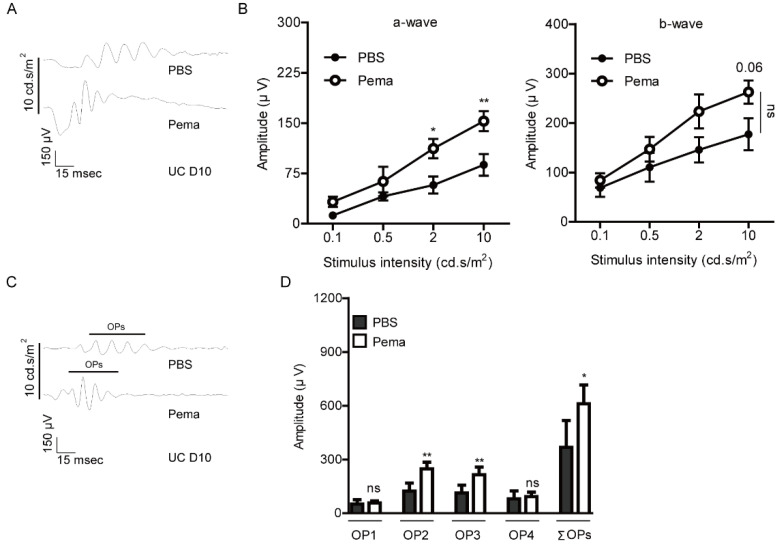
Protective effects of pemafibrate against retinal dysfunction 10 days after UCCAO. (**A**,**B**) Representative waveforms (10 cd·s/m^2^) of a-and b-waves and quantitative analyses (*n* = 5 per group) showed that oral administration of pemafibrate had a suppressing tendency in the reduction in the amplitudes of a-wave and b-wave in the UCCAO-operated eye 10 days after UCCAO with statistical significance. The data were analyzed using two-way ANOVA followed by a Bonferroni post hoc test (a-wave and b-wave). One datum was further analyzed using two-tailed Student’s *t*-test (b-wave; *p* = 0.06). The data were presented as mean ± standard error of the mean. (**C**,**D**) Representative waveforms (10 cd·s/m^2^) of oscillatory potentials (OPs) and quantitative analyses showed that pemafibrate significantly suppressed reduction in the amplitudes of OPs (OP1, OP2, OP3, and ΣOPs) in UCCAO-induced retinal ischemic mice (*n* = 5 per group). * *p* < 0.05, ** *p* < 0.01. The data were analyzed using two-tailed Student’s *t*-test. The data were presented as mean ± standard deviation. Pema; pemafibrate. UC; unilateral common carotid artery occlusion. ns; not significant.

**Figure A2:**
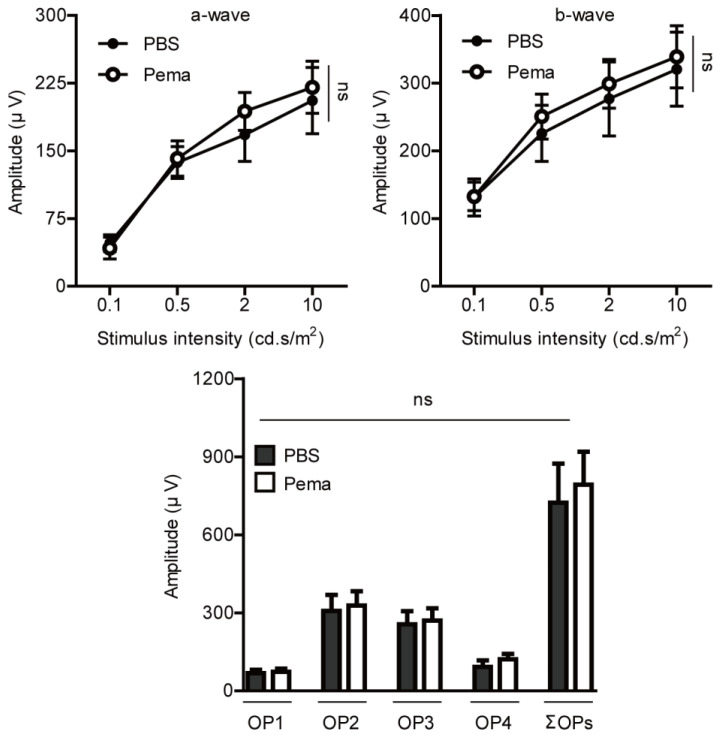
General measurements of retinal function for adult mice after consecutive oral administration of pemafibrate right before UCCAO. Quantitative analyses (*n* = 5 per group) showed that oral administration of pemafibrate had no effect on retinal function (a-wave, b-wave, and OPs) in adult naïve mice. *p* > 0.05. The data (a-and b-waves) were analyzed using two-way ANOVA followed by a Bonferroni post hoc test and presented as mean ± standard error of the mean. The data (OPs) were analyzed using two-tailed Student’s *t*-test and presented as mean ± standard deviation. Pema; pemafibrate, OPs; oscillatory potentials. ns; not significant.

Next, to investigate the protective effects of pemafibrate against retinal dysfunction in UCCAO-operated mice, electroretinography (ERG) was performed (Figure 1 and Figure 2). Before UCCAO, there was no significant difference in the amplitudes of a-and b-waves and the oscillatory potentials (OPs) between PBS-administered and pemafibrate-administered naïve mice (Figure A2). Previously, we demonstrated that retinal dysfunction was started from day 3 to day 7 after UCCAO [23,27]. Therefore, we primarily checked retinal dysfunction 5 days after UCCAO. We found that reduction in the amplitudes of a-and b-waves in UCCAO-operated mice was slightly suppressed by the oral administration of pemafibrate (Figure 1A,B). However, there was no statistical significance between PBS-administered UCCAO-operated mice and pemafibrate-administered UCCAO-operated mice. Next, we found that reduction in the amplitudes of OPs in UCCAO-operated mice was significantly suppressed by the oral administration of pemafibrate (Figure 1C,D).

Furthermore, reduction in the amplitudes of a-and b-waves in UCCAO-operated mice was slightly kept suppressed by the oral administration of pemafibrate 10 days after UCCAO (Figure 2A,B). Finally, we found that reduction in the amplitudes of OPs in UCCAO-operated mice was maintained to be suppressed by oral administration of pemafibrate (Figure 2C,D).

**Figure A3:**
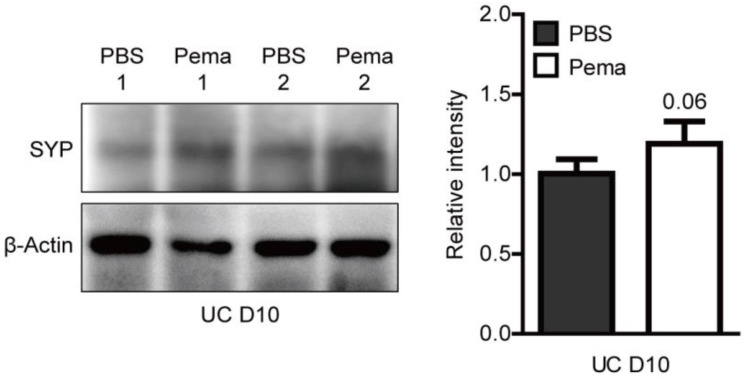
A slight increase in retinal synaptophysin (SYP) expression by oral administration of pemafibrate in UCCAO-operated mice. A representative image and quantitative analysis (*n* = 4 per group) showed that SYP expression slightly increased by oral administration of pemafibrate 10 days after UCCAO. *p* = 0.06. The data were analyzed using Student’s *t*-test and presented as mean ± standard deviation. Pema; pemafibrate, UC; unilateral common carotid artery occlusion.

Next, we examined the molecular mechanism underlying pemafibrate-mediated preservation of retinal function against UCCAO. Previously, we found that UCCAO decreased the expression of synaptophysin, one of the well-known synaptic vesicle proteins [27]. This protein is plentifully expressed in inner retinal neuronal cells which are the cellular source for OPs [34,35,36]. Even though there was no statistical significance, we found a decreasing synaptophysin expression after UCCAO was slightly suppressed in the pemafibrate-administered UCCAO-operated retina (Figure A3).

### 2.2. Suppression of Pathological Retinal Gliosis by Pemafibrate Administration in a Mouse Model of UCCAO-Induced Retinal Ischemia

**Figure 3:**
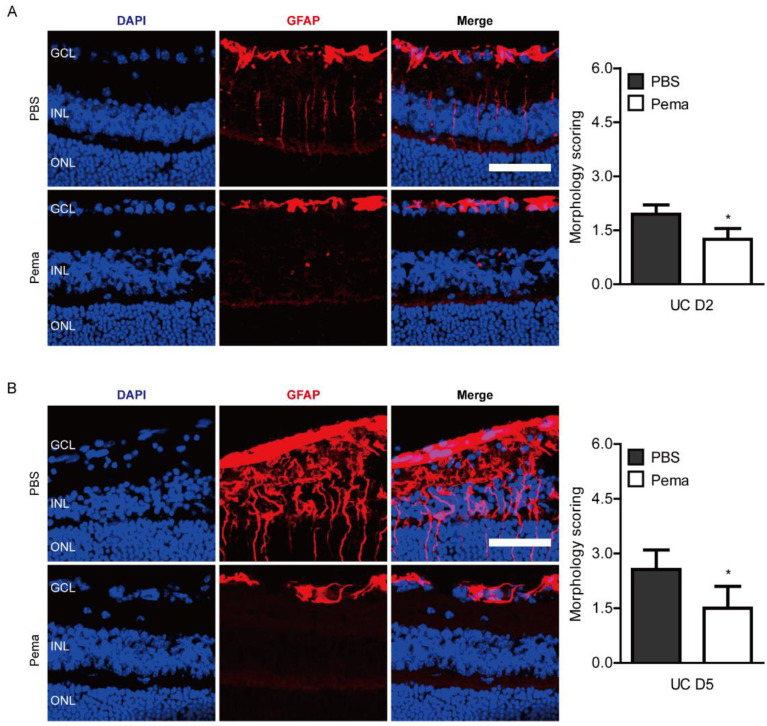
Modulation of pathological reactive gliosis after oral administration of pemafibrate. (**A**) Representative images and quantitative analyses (*n* = 4 per group) showed that slightly increased reactive retinal gliosis stained by GFAP in UCCAO-operated mice was reduced by administration of pemafibrate on day 2 after UCCAO. (**B**) Representative images and quantitative analyses (*n* = 4–5 per group) showed that dramatically increased reactive retinal gliosis stained by GFAP in UCCAO-operated mice were reduced by the administration of pemafibrate on day 5 after UCCAO. Scale bar: 50 µm. The data were analyzed using two-tailed Student’s *t*-test. Graphs were presented as mean with ± standard deviation. * *p* < 0.05. GCL: ganglion cell layer; INL: inner nuclear layer; ONL: outer nuclear layer. Pema; pemafibrate. UC; unilateral common carotid artery occlusion.

Reactive gliosis has been used as a responsive marker for retinal ischemic damages [37]. For further investigation of protective roles of pemafibrate against ischemic retinal dysfunction in UCCAO-operated mice, immunohistochemistry (IHC) was performed for detecting pathological reactive gliosis in the retina. Previously, we demonstrated that retinal gliosis was started from day 1 and more clearly seen on day 7 after UCCAO [17,23]. Therefore, we checked retinal gliosis from day 2 to day 5 after UCCAO (Figure 3). We found that slightly increased pathological glial responses on day 2 after UCCAO, observed by morphology scoring, were reduced in pemafibrate-administered UCCAO-operated mice (Figure 3A). Furthermore, as expected, dramatically increased pathological glial responses were seen 5 days after UCCAO, and these responses were significantly reduced in pemafibrate-administered UCCAO-operated mice (Figure 3B).

### 2.3. Screening of Hypoxia-Ischemia-Related Gene Expressions after Pemafibrate Administration in a Mouse Model of UCCAO-Induced Retinal Ischemia

**Figure 4:**
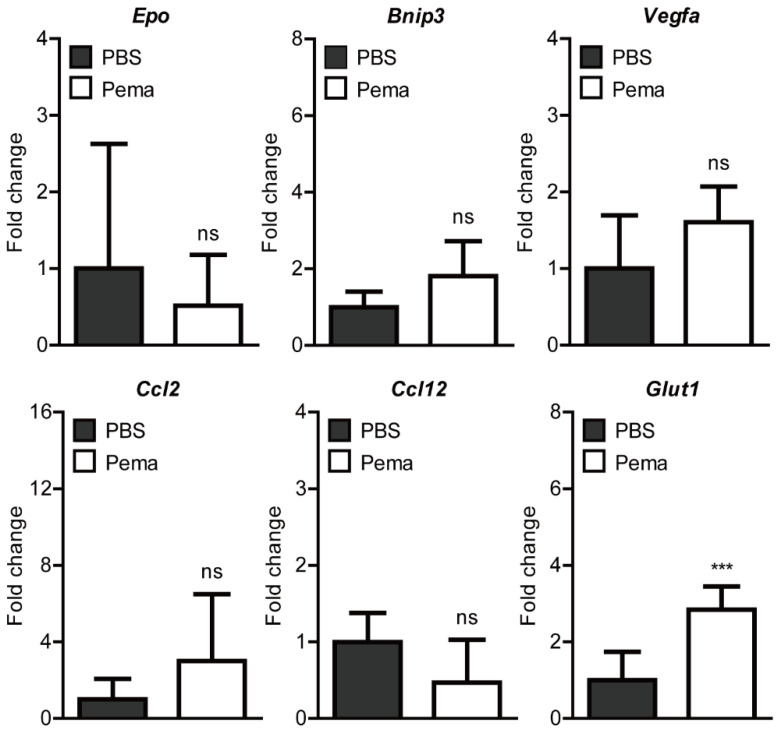
Screening of alterations in retinal hypoxia-ischemia-related gene expressions by oral administration of pemafibrate in UCCAO-operated mice. Primarily, genes reported to be slightly or dramatically altered after UCCAO were selected; *Epo*, *Bnip3*, *Vegfa*, *Ccl2*, *Ccl12*, and *Glut1*. Quantitative analyses (*n* = 6 per group) showed that oral administration of pemafibrate significantly reduced the expression of *Glut1* in the retina 1 day after UCCAO. However, the other genes’ expressions were not changed by oral administration of pemafibrate. *** *p* < 0.001. The data were analyzed using two-tailed Student’s *t*-test and presented as mean ± standard deviation. Pema; pemafibrate. ns; not significant.

Previously, it was reported that several hypoxia-ischemia-related gene expressions (*Epo*, *Bnip3*, *Vegfa*, *Ccl2*, *Ccl12*, and *Glut1*) were induced 1 day after UCCAO [17,27]. Therefore, we screened changes in these gene expressions after oral administration of pemafibrate (Figure 4). We found that the expression of *Glut1* significantly increased in the retina of pemafibrate-administered UCCAO-operated mice. Expressions of the other genes were not significantly altered by oral administration of pemafibrate.

### 2.4. Induction of PPARα Target Genes by Pemafibrate Administration in a Mouse Model of UCCAO-Induced Retinal Ischemia

**Figure 5:**
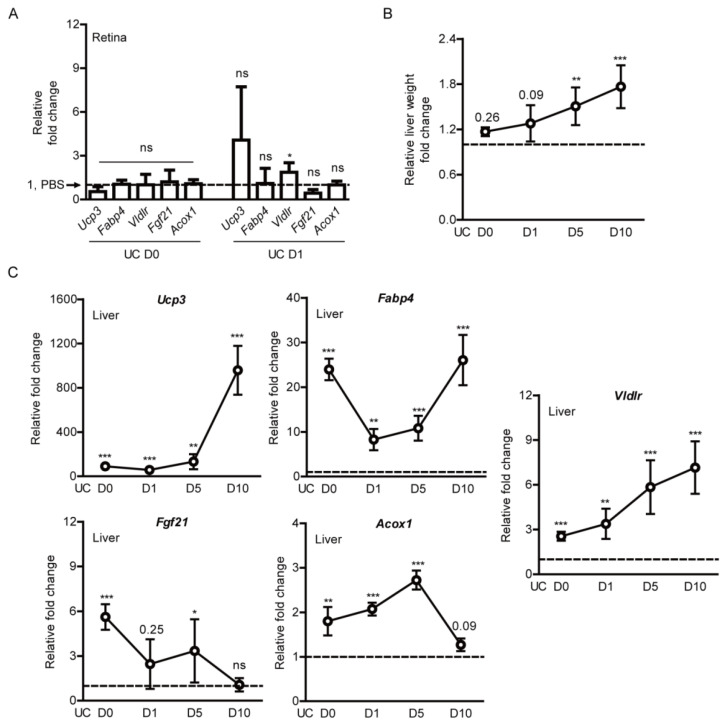
Induction in PPARα downstream gene expressions in the liver by oral administration of pemafibrate in UCCAO-operated mice. (**A**) Quantitative analyses (*n* = 4–6 per group) showed that oral administration of pemafibrate did not dramatically increase PPARα downstream gene expressions in the retina. The data were analyzed using two-tailed Student’s *t*-test and presented as mean ± standard deviation. (**B**) Quantitative analyses (*n* = 4–6 per group) showed that the relative liver weight (the liver weight/the body weight) in pemafibrate-administered mice was significantly higher than that in PBS-administered mice. The data were analyzed using two-tailed Student’s *t*-test and were presented as mean ± standard error of the mean. (**C**) Quantitative analyses (*n* = 4–5 per group) showed that oral administration of pemafibrate significantly increased PPARα downstream gene expressions in the liver. The data were analyzed using two-tailed Student’s *t*-test and presented as mean ± standard deviation. The value for PBS-administered mice was indicated as a dotted line; 1. * *p* < 0.05, ** *p* < 0.01, *** *p* < 0.001. Pema; pemafibrate, UC; unilateral common carotid artery occlusion. ns; not significant.

We examined whether expressions of PPARα downstream genes could be induced by the oral administration of pemafibrate. The retina was targeted as it is our primary region of interest. We could not find any significant change in *Ucp3*, *Fabp4*, *Vldlr*, *Fgf21*, and *Acox1* between the PBS-administered retina and the pemafibrate-administered retina on the day of UCCAO surgery (Figure 5A). Furthermore, we could not find any significant change in *Ucp3*, *Fabp4*, *Fgf21*, and *Acox1* 1 day after UCCAO. Even though a significant increase in *Vldlr* expression was detected, the fold change was not dramatic at all.

Next, the liver was targeted as it has been known as a region for pemafibrate-induced PPARα activation [24,38,39]. The livers in pemafibrate-administered UCCAO-operated mice seemed larger than those in PBS-administered UCCAO-operated mice while we collected the samples. Therefore, the liver weight was calculated with the body weight, and we found that the relative liver weight gradually increased after consecutive administration of pemafibrate with statistical significance, in comparison with that in PBS-administered UCCAO-operated mice (Figure 5B). Furthermore, PPARα downstream genes (*Ucp3*, *Fabp4*, *Vldlr*, *Fgf21*, and *Acox1*) in the liver increased significantly and dramatically, in comparison with those in PBS-administered UCCAO-operated mice (Figure 5C). Especially, two genes (*Ucp3* and *Vldlr*) were gradually increased in a time-dependent manner. Even though there was fluctuation, *Fabp4* was also time-dependently increased after long-term repetitive oral administration of pemafibrate. When it comes to *Fgf21*, its gene expression was dramatically induced at the early stage of repetitive oral administration of pemafibrate and gradually decreased after long-term repetitive administration of pemafibrate. There was a gradual increasing tendency in the expression of *Acox1* until 5 days after UCCAO, and its expression was detected to the basal level on day 10 after UCCAO.

**Figure 6:**
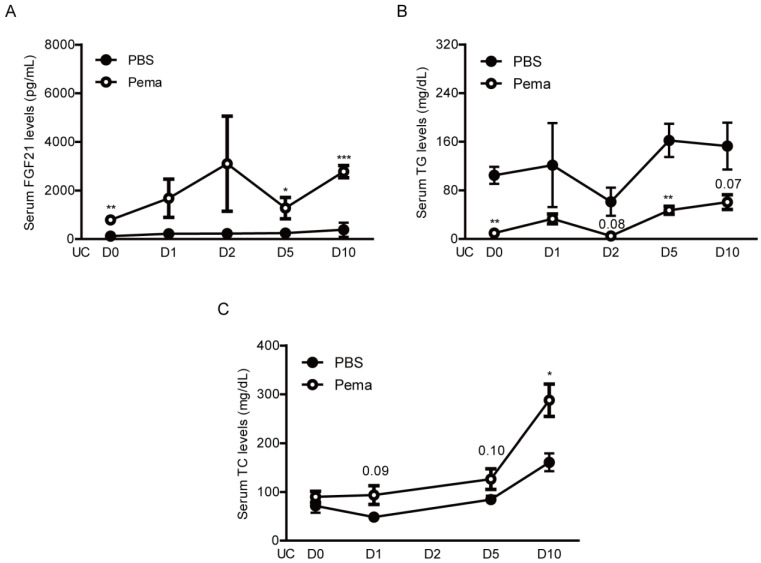
Changes in serum levels of FGF21, TG, and TC by oral administration of pemafibrate in UCCAO-operated mice. (**A**) Quantitative analyses (*n* = 3–9 per group) showed that oral administration of pemafibrate significantly increased serum levels of FGF21. The data were analyzed using two-tailed Student’s *t*-test and presented as mean ± standard error of the mean. (**B**,**C**) Quantitative analyses (*n* = 3–8 per group) showed that oral administration of pemafibrate significantly decreased serum levels of TG and increased serum levels of TC. The data were analyzed using two-tailed Student’s *t*-test and presented as mean ± standard error of the mean. * *p* < 0.05, ** *p* < 0.01, *** *p* < 0.001. Pema; pemafibrate, UC; unilateral common carotid artery occlusion, TG; triglyceride, TC; total cholesterol.

Moreover, we examined whether pemafibrate is able to increase serum levels of FGF21. Increases in serum FGF21 levels by PPARα agonists have been reported in various experimental models and clinical studies [27,30,31,40,41,42]. As expected, elevated serum levels of FGF21 were dramatically seen after oral administration of pemafibrate on the day of the UCCAO surgery (Figure 6A). Furthermore, increased serum FGF21 levels were continuously observed in pemafibrate-administered UCCAO-operated mice until the end of experiments, in comparison with PBS-administered UCCAO-operated mice. 

Next, triglyceride (TG) and total cholesterol (TC) levels in the serum were examined (Figure 6B,C), as TG and TC levels have also been reported to be changed by administration of pemafibrate in various experimental models and clinical studies [31,43,44,45]. Expectedly, we detected continuous decreases in serum TG levels and increases in serum TC levels in pemafibrate-administered UCCAO-operated mice in comparison with PBS-administered UCCAO-operated mice.

### 2.5. Observation of Retinal Thickness Changes by Pemafibrate Administration in a Mouse Model of UCCAO-Induced Retinal Ischemia

**Figure A4:**
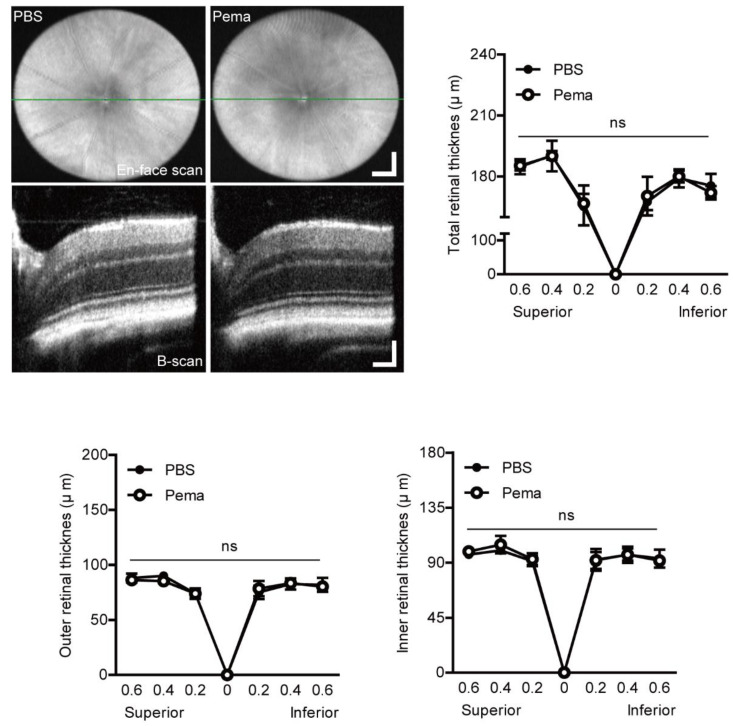
No alteration in retinal thickness by oral administration of pemafibrate in UCCAO-operated mice. Representative OCT images (b-scan) in the PBS-and pemafibrate-administered UCCAO-operated retinas and quantitative analyses (*n* = 5 per group) showed that there was no change in retinal thickness (total, outer, and inner retinal layers) on day 10 after UCCAO. The values in the horizontal axis of the graph stand for 0.2, 0.4, and 0.6 mm distance from the optic nerve head (0) that was detected by the green line (en-face scan). Representative OCT images were taken at 0.4 mm from the optic nerve head. The data were analyzed using two-way ANOVA followed by a Bonferroni post hoc test and presented as a spider diagram (mean ± standard deviation). *p* > 0.05. Scale bars are 200 (en-face scan; vertical and horizontal bars) and 200 and 100 (b-scan; vertical and horizontal bars) µm, respectively. Pema; pemafibrate, OCT; optical coherence tomography. ns; not significant.

Previously, we could not find changes in retinal thickness 14 days after UCCAO [17,23]. In case of unknown effects of pemafibrate on retinal thickness, we examined whether retinal thickness could be changed by the oral administration of pemafibrate using optical coherence tomography (OCT) (Figure A4). Expectedly, we could not find any particular change in the retina and retinal thickness after oral administration of pemafibrate on day 10 after UCCAO.

## 3. Discussion

We revealed that the consecutive oral administration of pemafibrate, a selective PPARα modulator, suppressed pathological retinal gliosis and functional neuronal deficits in a murine model of retinal ischemia by UCCAO. Furthermore, we found significant increases in PPARα target gene expressions in the liver, not in the retina, reduction in serum levels of TG, and elevation in serum levels of FGF21 and TC. Previous single-cell data demonstrated that PPARα had low expression in the retina [46]. On the other hand, FGFR1, a crucial receptor for FGF21 function, was highly expressed in several types of cells in the retina. That could be the reason that pemafibrate did not activate PPARα extensively in the retina. On the other hand, it is reported that PPARα is a key modulator of hepatic FGF21 [47]. This is consistent with our previous reports and other studies that PPARα agonists (pemafibrate or fenofibrate) increase serum levels of FGF21 as well as boost liver function to exert therapeutic effects in ischemic retinopathies such as diabetic retinopathy or ocular ischemic syndrome [27,30,31,48]. 

FGF21 comprises 209 amino acids, and its protein regulates critical metabolic pathways [49,50,51,52]. FGF21 is produced in various tissues, especially in the liver [52], and improves lipid profiles in patients with type 2 diabetes [53]. So far, several studies have shown FGF21’s therapeutic roles in the retina in vitro and in vivo (Table 1). Fu et al. showed that long-acting FGF21 suppressed neovascularization in mice by suppressing TNF-α expression via increasing adiponectin levels [54]. They also showed that long-acting FGF21 preserved retinal function (analyzed using ERG) in streptozotocin-induced diabetic mice and Akita mice which mimic type 1 diabetes [55]. Additionally, they showed that FGF21 suppressed oxidative stress-induced inflammation in 661W cells. Our group showed that long-acting FGF21 reduced retinal vascular leakage in a murine model of retinal vascular leakage and demonstrated that long-acting FGF21 maintained claudin-1 expression in human endothelial cells [56]. We recently reported that long-acting FGF21 improved retinal neuronal function through Müller glial remodeling in P23H mice, studied along with in vitro rat retinal Müller glial cells [57]. On top of that, we showed that pemafibrate showed therapeutic effects against pathological neovascularization in a murine model of oxygen-induced retinopathy and rescued retinal function in streptozotocin-induced diabetic mice through increasing FGF21 levels in the blood [30,31]. Taken together, increases in serum FGF21 levels induced by pemafibrate administration may also have the same protective effects on the UCCAO-induced ischemic retina. However, further studies are needed to see if direct FGF21 injection could exert cellular protection in the ischemic retina.

Based on our current data, an increase in *Glut1* expression was seen in the pemafibrate-administered UCCAO-induced ischemic retina. Previously, we also demonstrated that *Glut1* expression increased in the retina of the same ischemic murine model after consecutive oral administration of fenofibrate, a well-known PPARα agonist [27]. We assume that elevation in serum levels of FGF21 is one of the reasons for the induction of *Glut1* in the retina. FGF21 has been suggested to exert a therapeutic effect on glucose and lipid metabolisms in mice [58]. Regarding this effect, a clinical trial has been studied using a novel long-acting FGF21 pegbelfermin which may have therapeutic effects on nonalcoholic fatty liver disease and nonalcoholic steatohepatitis [24]. Previously, FGF21 showed a synergistic effect with insulin on glucose absorption associated with an enhancement in *Glut1* expression [59]. FGF21 could regulate glucose and lipid metabolisms through the induction of FGF21 downstream signaling molecules including *Glut1* [60,61]. In adipocytes, an increase in *Glut1* mRNA expression has been along with upregulation of FGF21 [62]. Moreover, cardiac protection by administering FGF21 against ischemia/reperfusion-induced cardiac damages has been explained by the upregulation of GLUT1 [63]. Suppressed *Glut1* expression may impair an entry of glucose into photoreceptors, which results in a lack of lipid and glucose fuel for retinal function [64]. In this regard, elevated serum levels of FGF21 may support the induction of *Glut1*. This effect may bring positive outcomes to the damaging retina under acute hypoperfused states through modulation of glucose metabolism. However, previous reports suggested that the suppression of diabetic retinopathy could be involved with GLUT1 inhibition [65,66]. There may have a discrepancy between experimental models of our OIS and diabetic retinopathy in that blood glucose levels between them are totally different and the duration of diseases are not the same either. In fact, controversial reports on GLUT1 expression in diabetic retinopathy itself already exist. In the retina and its microvessels of streptozotocin-induced diabetes, downregulated GLUT1 expression was detected [67]. As determined by GLUT1 immunogold staining, compensatory downregulation of GLUT1 on the inner blood-retinal barrier was not seen in diabetic rats [68]. Chronic hyperglycemia led to a decrease in GLUT1 protein expression without alteration in its mRNA expression in the retina of diabetic Goto Kakizaki rats and alloxan-treated diabetic rabbits [69]. Taken together, more studies are needed for understanding the potential role of GLUT1 depending on the disease states.

**Figure A5:**
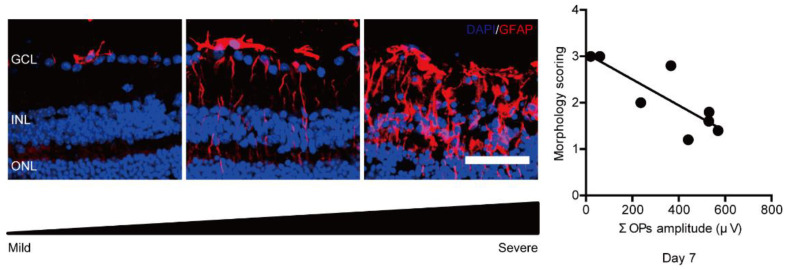
A relationship between pathological retinal gliosis and retinal dysfunction 7 days after UCCAO. Representative images of morphology scoring for pathological retinal gliosis (acquired from our preliminary experiments) and visualization in a correlation between pathological retinal gliosis and the amplitudes of ΣOPs showed that there was a high correlation between pathological gliosis and loss of the amplitudes of ΣOPs in the UCCAO-operated eye, determined by regression analyses. Dots (*n* = 8) represent each morphology scoring and the amplitude of ΣOPs. A line represents the linear fit of the data points. Scale bar: 50 µm. Slope: −0.0028; Y-intercept: 3.06; r^2^: 0.68. *p* < 0.05. GCL: ganglion cell layer; INL: inner nuclear layer; ONL: outer nuclear layer, UC; unilateral common carotid artery occlusion.

It is reported that gliosis and loss of the amplitudes of OPs could be a hallmark of the early phase in a streptozotocin-induced diabetic mouse model and OIS mouse models [14,23,70]. Based on our preliminary data, there was a high correlation between pathological gliosis and loss of the amplitudes of OPs in the UCCAO-operated eye (Figure A5). This implies that retinal functional deficits may be along with the induction of pathological gliosis. Previously, fenofibrate modulated pathological gliosis and improved ERG abnormalities in *db*/*db* mice [48,71]. Similarly, pemafibrate modulated pathological gliosis and preserved retinal function in the ischemic retina based on our current data. In fact, we previously reported that pemafibrate maintained the amplitudes of OPs in a murine model of diabetes via maintaining the expression of synaptophysin, a marker of synapse [31]. Even though the expression of synaptophysin had a slight increase by pemafibrate administration, we assume that our current results have a consistency with the results in our previous study [31] and other PPARα studies [48,71].

In our current research, pemafibrate maintained the amplitude of a-wave in the UCCAO model, which indicates that the function of photoreceptors was protected by pemafibrate administration [72,73]. Müller glial cells have an important role in maintaining photoreceptors as well as retinal pigment epithelium [74]. We recently reported that FGF21 preserved photoreceptor function via modulating Müller glial cells in P23H mice which mimic human retinitis pigmentosa [57]. Additionally, FGF21 increased the synapse formation pathway in the retina and induced Müller glial axon development genes. The synaptic connection between the inner retina and the outer retina was also preserved by FGF21 treatment. Taken together, pemafibrate may have the potential to rescue inner and outer retinal cells via modulating Müller glial cells, observed by the preservation of OPs and a-wave. However, we need further studies to clarify the mechanism. On the other hand, pemafibrate did not affect retinal thickness as seen in our current data. In fact, we did not observe dramatic changes in retinal thickness in UCCAO-operated mice in comparison with that in sham-operated mice [23]. Taken together, pemafibrate could primarily influence retinal function, which is consistent with our previous report [31].

In our study, reduced levels of TG were seen after administration of pemafibrate. Furthermore, increased levels of TC (speculated as HDL-C [31,44,75]) were shown after the administration of pemafibrate. High levels of TG are suggested as one of the risk factors in human cardiovascular diseases [76,77,78]. Furthermore, it was reported that the TG/HDL-C ratio was highly associated with an increased risk of developing retinopathy [79,80]. It has also been reported that the high TG/HDL-C ratio could act on endothelial dysfunction, chronic low-grade inflammation, and coagulation [79,81]. Although the experimental UCCAO mouse model may not have dramatic metabolic stresses systemically, high levels of TG may exacerbate stenosis of CCA in human metabolic cardiovascular disease states, and pemafibrate could have preventive and protective roles on the stenosis of CCA via decreasing high TG levels in the blood. Similarly, metabolic changes could be considered as important factors in the development of retinal diseases [82,83]. Although we did not deeply cover systemic metabolic changes by the administration of pemafibrate, PPARα activation has been suggested as a regulator of β-oxidation [38,84]. WY16463, one of the selective PPARα agonists, showed reducing effects on the number of retinal angiomatous proliferation-like vascular lesions in the *Vldlr*−/− retina via the possible mechanism of enhancement of β-oxidation [64]. Pemafibrate also has been suggested to enhance β-oxidation [85,86]. Taken together, we speculate that more therapeutic effects of pemafibrate could be seen if we develop a new murine model of retinal ischemia by UCCAO in metabolic disorder models (which are more clinically relevant) and treat pemafibrate in those ischemic retinas. This will be further studied.

In the current study, the oral administration of pemafibrate was tested. Even though various methods for drug administration such as intraperitoneal injection, or intravitreal injection could be tested in our UCCAO model, we believe that our current method is patient-friendly (in terms of repetitive administrations of pemafibrate) and pain-free in the eye or body (as it is a non-invasive procedure) [87].

Now, Pemafibrate to Reduce Cardiovascular OutcoMes by Reducing Triglycerides IN patiENts With diabeTes (PROMINENT) study in patients with type 2 diabetes mellitus and dyslipidemia is undergoing all over the world (ClinicalTrials.gov Identifier: NCT03071692, accessed on 20 July 2021). Unfortunately, PROMINENT eye study, which tried to evaluate patient with diabetic retinopathy, was terminated because of a lack of recruited patients. Another clinical trial for pemafibrate has been completed for nonalcoholic fatty liver disease (NAFLD) and clinical scientists are waiting for the results (ClinicalTrials.gov Identifier: NCT03350165, phase 2, accessed on 20 July 2021). If the positive effect of pemafibrate on cardiovascular diseases or NAFLD could be seen, pemafibrate might have chances to be repositioned for retinal diseases in the future.

**Figure 7:**
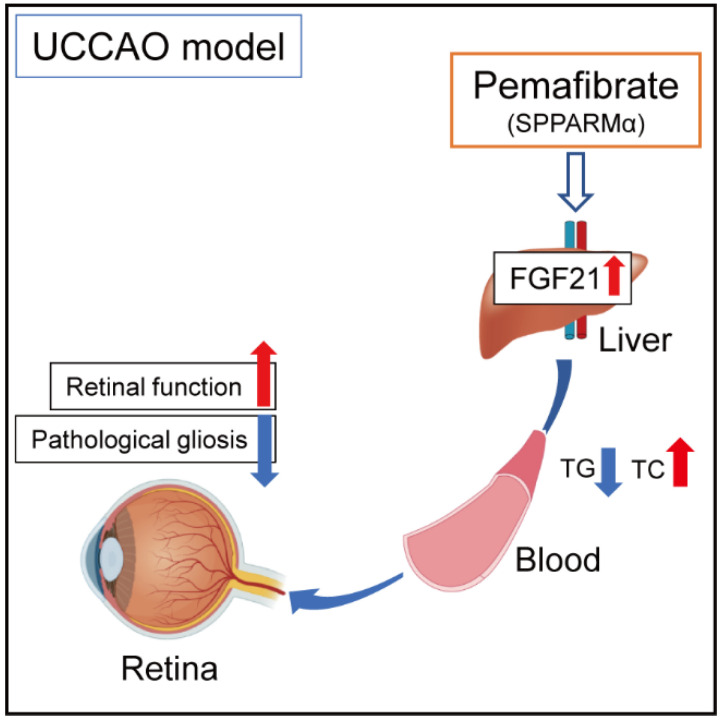
A working hypothesis of the protective mechanism against retinal dysfunction by administering pemafibrate in a murine model of retinal ischemia by UCCAO. The possible mechanisms for suppression of retinal dysfunction induced in cardiovascular diseases are that consecutive administration of systemic selective PPARα modulator (SPPARMα) pemafibrate enhances liver function and upregulates PPARα target genes in the liver, and elevated levels of serum FGF21 (one of the strong neuroprotective agents) modulate pathological gliosis and maintain the amplitudes of OPs. Indirectly, a reduction in levels of TG and an induction in levels of TC may have a risk-decreasing effect on developing retinopathy in humans. TG; triglyceride, TC; total cholesterol.

In conclusion, even though we need more links regarding retinal protection by activating PPARα in the liver, we suggest a promising pemafibrate therapy in carotid artery occlusion-induced ischemic retinopathy, with boosting liver function, regulating serum levels of FGF21, TC, and TG, and suppressing retinal dysfunction (Figure 7).

## 4. Materials and Methods

### 4.1. Animal

A number of 6–8 weeks old male C57BL/6 mice were obtained from CLEA Japan (Tokyo, Japan) and supplied freely with food and water under a twelve-hour light-dark cycle in a temperature-managed room. All protocols were permitted by the Ethics Committee on Animal Research of the Keio University School of Medicine (Approved number #16017/2020). All procedures complied with the ARVO Statement for the Use of Animals in Ophthalmic and Vision Research and the international standards of animal care and use, Animal Research: Reporting in Vivo Experiments (ARRIVE) guidelines (accessed on 20 July 2021, http://www.nc3rs.org.uk/arrive-guidelines). 

### 4.2. A Murine Model of UCCAO-Induced Retinal Ischemia and Oral Administration of Pemafibrate

Randomized mice were orally provided 0.5% DMSO-dissolved PBS or pemafibrate (0.5 mg/kg in 0.5% DMSO-dissolved PBS) for four days daily before UCCAO. A mouse model of UCCAO-induced retinal ischemia was induced, as previously described [17]. Briefly, deep anesthesia was induced to mice with a combination of midazolam (40 μg/100 μL; Sandoz, Tokyo, Japan), medetomidine (7.5 μg/100 μL; Orion, Espoo, Finland), and butorphanol tartrate (50 μg/100 μL; Meiji Seika Pharma, Tokyo, Japan) [22]. The mouse neck was incised to observe the common carotid artery. Then, the common carotid artery in the right side was permanently occluded using 6–0 silk sutures. Wounds of the neck were clearly sutured, and the mouse was recovered. Pemafibrate was continuously supplied to mice daily 1 day after UCCAO until the end of experiments. The body weight was measured during the whole experimental period, and the liver weight was measured on the day of sample collection.

### 4.3. Optical Coherence Tomography (OCT)

OCT (Envisu R4310, Leica, Wetzlar, Germany) was conducted as previously described [22,27]. Briefly, mice were subjected to mydriasis by a combination of 0.5% tropicamide and 0.5% phenylephrine (Santen Pharmaceutical, Osaka, Japan). After 5 min, mice were anesthetized as same as Section 4.2. Anesthetized mice were quickly subjected to OCT analyses. B-scan images were obtained from equatorial slices of en-face scans, and images in 0.2, 0.4, and 0.6 mm from the optic nerve head were taken. Retinal thickness was measured from the outer retina to the inner retina as we described [27].

### 4.4. Electroretinography (ERG)

ERG was conducted as previously described [27]. Briefly, mice were placed for more than 12 h for dark adaptation. Pupils were dilated as Section 4.3. Mice were anesthetized as Section 4.2 after 5 min incubation. Recording of scotopic ERG responses was processed using a Ganzfeld dome and LED stimulators with an acquisition system (PuREC, MAYO, Inazawa, Japan). The amplitudes of a-wave and b-wave were measured with various light stimuli. Furthermore, the amplitudes of OPs were measured at the four peaks of OPs as previously described [23].

### 4.5. Immunohistochemistry (IHC)

IHC was performed as previously [23]. Briefly, eyes were fixed with PFA (4%), and O.C.T. Compound (Sakura Tissue-Tek, Tokyo, Japan) was applied to embed the eyes for frozen sectioning. The sagittal sectioning slides using Cryostat (Leica CM3050S, Leica, Wetzlar, Germany) were incubated in a blocking solution (PBS + 0.1% Triton + 0.1% BSA). Then, a primary antibody (GFAP 1:400, Cat #13-0300, Thermo Fisher Scientific, Waltham, MA, USA) was added to the eyes. The eyes were washed with PBS + 0.1% Triton and soaked into a solution of a species-appropriate fluorescence-conjugated secondary antibody (Thermo Fisher Scientific, Waltham, MA, USA) for several hours. After washing with PBS + 0.1% Triton three times, DAPI was shortly incubated. After washing with PBS again, the eyes were mounted and examined via a fluorescence microscope (LSM710, Carl Zeiss, Jena, Germany), as previously described [22]. The fluorescence immunoreactivity was quantified by a morphology score as previously described [12,17,23] with a minor modification: 0 = no signal, 1 = labeled processes in the ganglion cell layer, 2 = weakly labeled processes in the inner retinal layer, including the ganglion cell layer, and 3 = strongly labeled processes in the entire retinal layer including the inner and outer retinas.

### 4.6. Measurement of Serum FGF21, TC, and TG Levels

After blood collection and serum extraction as previously described [27,31], serum samples were evaluated with an FGF21 ELISA kit (Cat #RD291108200R, BioVendor Laboratory Medicine, Brno, Czech Republic), a TC kit (Cat #STA-384, Cell Biolabs, Inc., San Diego, CA, USA), and a TG kit (Cat #STA-396, Cell Biolabs, Inc., San Diego, CA, USA) following the manufacturer’s instructions.

### 4.7. Quantitative PCR

Quantitative PCR was conducted, as previously described [27]. Briefly, the retina and the liver mRNA were extracted using an RNeasy Plus Mini Kit (Qiagen, Venlo, The Netherlands). RT-PCR was conducted with a ReverTra Ace^®^ qPCR RT Master Mix with gDNA Remover (TOYOBO, Osaka, Japan). Quantitative PCR was conducted using a THUNDERBIRD^®^ SYBR^®^ qPCR Mix (TOYOBO, Osaka, Japan) with the Step One Plus Real-Time PCR system (Applied Biosystems, Waltham, MA, USA). The primers that we used are entered in Table 2. The fold alteration between levels of different transcripts was calculated by the ΔΔCT protocol.

### 4.8. Western Blotting

Western Blotting was conducted as described in our previous paper [27]. We used anti-synaptophysin (1:1000, Cat #SAB4502906, Sigma, Tokyo, Japan) and anti-β-Actin (1:5000, #3700, Cell Signaling Technology, Danvers, MA, USA). After incubation of primary antibodies, HRP-conjugated secondary antibodies (1:1000 for anti-synaptophysin; 1:5000 for anti-β-Actin, GE Healthcare, Chicago, IL, USA) were put to the membrane. Intensities of the bands were quantified via NIH ImageJ program (National Institutes of Health, Bethesda, MD, USA).

### 4.9. Statistical Analysis

Data were analyzed with GraphPad Prism 5 (GraphPad Program, San Diego, CA, USA) and calculated by using a two-way Student’s *t*-test or two-way ANOVA followed by a Bonferroni post hoc test depending on the dataset. Any *p*-values of less than 0.05 were regarded as statistically significant.

## Figures and Tables

**Table 1:** Therapeutic Roles of FGF21 in the Eye (studied using PF-05231023, a long-acting FGF21 analog).

Author	Year of Publication	Journal	In Vitro	Effect	In Vivo	Effect
			Cell Type		Experimental Model	
Fu et al. [54]	2017	*Cell Reports*	HRMEC	Promotes cell migration	OIR; VldlrKO; Laser-induced CNV	Suppresses NV via decreasing TNF-α expression
Fu et al. [55]	2018	*Diabetes*	661 W	Inhibits oxidative stress-induced inflammation	STZ; Akita mouse	Rescues retinal morphology and function
Tomita and Ozawa et al. [30]	2019	*IJMS*	661 W	Inhibits a HIF activity	-	-
Tomita et al. [56]	2020	*IJMS*	HRMEC	Prevents vascular permeability	mVEGF164-induced retinal vascular leakage mouse	Preserves an expression of tight junction protein
Tomita and Lee et al. [31]	2020	*IJMS*	PC12D	Increases synaptophysin protein expression	-	-
Fu and Qiu et al. [57]	2021	*iScience*	rMC-1	Increases SRF protein expression	P23H mutation mouse	Modulates retinal glial responses

HRMEC: human retinal microvascular endothelial cell; 661W: cone photoreceptor cell; PC12D: pheochromocytoma 12D neuronal cells; rMC-1: rat retinal Müller glia; HIF: hypoxia-inducible factor; SRF: serum response factor; OIR: oxygen-induced retinopathy; Vldlr KO: very-low-density lipoprotein receptor knock out; CNV: choroidal neovascularization; NV: neovascularization; STZ: streptozotocin-induced diabetes; mVEGF164: mouse vascular endothelial growth factor 164.

**Table 2:** Primer list.

Name	Direction	Sequence (5′ → 3′)	Accession Number
*Hprt*	Forward	TCAGTCAACGGGGGACATAAA	NM_013556.2
	Reverse	GGGGCTGTACTGCTTAACCAG	
*Epo*	Forward	GGCCATAGAAGTTTGGCAAG	NM_007942
	Reverse	CCTCTCCCGTGTACAGCTTC	
*Bnip3*	Forward	GCTCCCAGACACCACAAGAT	NM_009760.4
	Reverse	TGAGAGTAGCTGTGCGCTTC	
*Vegfa*	Forward	CCTGGTGGACATCTTCCAGGAGTACC	AY707864.1
	Reverse	GAAGCTCATCTCTCCTATGTGCTGGC	
*Glut1*	Forward	CAGTTCGGCTATAACACTGGTG	NM_011400.3
	Reverse	GCCCCCGACAGAGAAGATG	
*Ccl2*	Forward	CCCAATGAGTAGGCTGGAGA	NM_011333.3
	Reverse	TCTGGACCCATTCCTTCTTG	
*Ccl12*	Forward	GCTACAGGAGAATCACAAGCAGC	NM_011331.3
	Reverse	ACGTCTTATCCAAGTGGTTTATGG	
*Ucp3*	Forward	GGAGTCTCACCTGTTTACTGACAACT	NM_009464.3
	Reverse	GCACAGAAGCCAGCTCCAA	
*Fabp4*	Forward	CCGCAGACGACAGGA	NM_024406.3
	Reverse	CTCATGCCCTTTCATAAACT	
*Fgf21*	Forward	AACAGCCATTCACTTTGCCTGAGC	NM_020013.4
	Reverse	GGCAGCTGGAATTGTGTTCTGACT	
*Vldlr*	Forward	GAGCCCCTGAAGGAATGCC	NM_001161420.1
	Reverse	CCTATAACTAGGTCTTTGCAGATATGG	
*Acox1*	Forward	TCTTCTTGAGACAGGGCCCAG	AF006688.1
	Reverse	GTTCCGACTAGCCAGGCATG	

## Data Availability

The data presented in this study are available on request from the corresponding author.
